# Establishing an OCD Model in BALB/c Mice Using RU24969: A Molecular and Behavioural Study of Optimal Dose Selection

**DOI:** 10.1155/2024/4504858

**Published:** 2024-03-25

**Authors:** Fatima Salloum, Mohamad Farran, Houssam Shaib, Abdo Jurjus, Roni Sleiman, Mahmoud I. Khalil

**Affiliations:** ^1^Department of Biological Sciences, Faculty of Science, Beirut Arab University, Beirut, Lebanon; ^2^Department of Agriculture, Faculty of Agricultural and Food Sciences, American University of Beirut, Beirut, Lebanon; ^3^Department of Anatomy, Cell Biology, and Physiology, Faculty of Medicine, American University of Beirut, Beirut, Lebanon; ^4^Molecular Biology Unit, Department of Zoology, Faculty of Science, Alexandria University, Alexandria, Egypt

## Abstract

Obsessive-compulsive disorder (OCD) is a disabling disease characterized by distressing obsessions and repetitive compulsions. The etiology of OCD is poorly known, and mouse modeling allows to clarify the genetic and neurochemical basis of this disorder and to investigate potential treatments. This study evaluates the impact of the 5-HT1B agonist RU24969 on the induction of OCD-like behaviours in female BALB/c mice (*n* = 30), distributed across five groups receiving varying doses of RU24969. Behavioural assessments, including marble test, tail suspension test, sucrose preference test, forced swim test, and nestlet shredding test, were conducted. Gene expression and protein quantitation of Gabra1 and serotonin transporter in mouse brain were also performed. Marble-burying behaviour increased significantly at high doses of RU24969 (15-20 mg/kg). The forced swimming test consistently showed elevated values at the same high concentrations, compared to the control. Altered reward-seeking behaviour was indicated by the sucrose preference test, notably at 15 and 20 mg/kg doses of RU24969. Nestlet shredding results did not show statistical significance among the tested animal groups. Gene expression analysis revealed reduced Gabra1 expression with increasing doses of RU, while serotonin transporter was not related to varying doses of RU24969. Western blotting corroborated these trends. The results underscore complex interactions between the serotonin system, GABAergic signaling, and OCD-relevant behaviours and suggest the use of intraperitoneal injection of 15 mg/kg of RU24969 to induce OCD-like behaviour in BALB/c mouse models.

## 1. Introduction

Obsessive-compulsive disorder (OCD) is a mental health condition that affects individuals of various ages and backgrounds. It occurs when a person becomes trapped in a repetitive cycle of obsessions and compulsions. Obsessions refer to unwanted and intrusive thoughts, images, or urges that trigger intense distress [1]. On the other hand, compulsions are actions taken by individuals in an attempt to alleviate their obsessions and reduce their levels of distress. The precise cause of OCD is not definitely known, but it is influenced by many factors such as genetics, environment, personality traits, and stressors. Many studies provided strong evidence that the transmission of OCD is consistent with genetic transmission through specific brain chemical expression. These brain chemicals, such as serotonin, glutamate, and brain-derived neurotrophic factor, are involved in the severity of OCD symptoms [2–5]. Another factor, which is the environment, can affect the initiation and/or magnitude of OCD symptoms. For instance, stressful or traumatic events and family problems can worsen OCD symptoms, especially for people who already have genetic predisposition. Infections and illnesses, such as strep throat, can also change the brain chemistry and immune system and might trigger OCD-like behaviour. A third factor is personality traits, which are aspects of a person's character that are stable and consistent. Some personality traits, such as being very emotional, very cautious, or very perfectionist, may make a person more likely to develop OCD symptoms [6]. It is important to note that there is no single psychological factor that can be pinpointed as the sole cause of OCD in any given individual [6].

The serotonin system has traditionally played a significant role when studying OCD using mouse models. Researchers have utilized transgenic and optogenetic tools, alongside classic pharmacology and behavioural techniques, in mouse models to advance the understanding of underlying pathological processes. While studies involving human OCD patients provide some insights into the disease, their noninvasive nature limits the ability to dissect the pathological mechanisms [6, 7]. However, the emergence of strategies for genetic and circuit-specific manipulation in mouse models has allowed researchers to identify the molecular and cellular events associated with abnormal repetitive behaviour and in relation to OCD [6].

Two major mouse models involving pharmacologic stimulation have been investigated in relation to abnormal repetitive behaviours observed in OCD. These models are quinpirole-induced checking and administration of serotonin-1B receptor (5-HT1B) agonists, such as RU24969 [6]. Studies have explored the potential connection between 5-HT1B stimulation and the development of abnormal repetitive behaviours. Researchers discovered that the injection of a 5-HT1B agonist resulted in both perseverative locomotion and deficits in prepulse inhibition, which are observed in patients with OCD. However, these effects were reversed with chronic fluoxetine treatment, but not with acute treatment. A subsequent study localized the responsible receptors in the orbitofrontal cortex (OFC), drawing parallels to the circuit abnormalities seen in patients with OCD [8, 9]. Furthermore, others showed that 5-HT1B agonist treatment also led to impaired performance in the delayed alternation task, similar to impaired performance observed in patients with OCD [6–11].

The aim of the present study is to develop an OCD mouse model using RU24969, a 5-HT1B agonist, with specific concentrations of 0, 5, 10, 15, and 20 mg/kg body weight. In contrast to existing studies, our research explores either a broader range of RU24969 doses, a different increment interval (5 mg/kg), or a different mouse strain. This extended dose spectrum is crucial for capturing a comprehensive understanding of the behavioural traits associated with compulsive phenotype in mice [10–14].

A set of behavioural tests coupled with specific gene and protein expression analysis were used to provide a more accurate prediction of the outcomes in the developed model.

## 2. Materials and Methods

### 2.1. Experimental Groups

A total of 30 two-month-old female BALB/c mice weighing each around 20-25 g were equally divided into five groups of 6 mice each. All animals were housed in psychopharmacology laboratory at the Animal Facilities of the American University of Beirut under controlled temperature in a natural light-dark cycle of 12 hrs each. The animals were kept in cages containing wood shavings and have unrestricted access to a commercial mouse diet (24% protein, 4.5% fat, and 4% fibers) and water [15]. Mice were kept in groups of 6 mice/cage for a two-week adaptation period and then individually caged at the start of the modeling process. The 5-HT1B agonist RU24969 (MCE, 1 Deer Park Dr, Suite Q, Monmouth Junction, NJ 08852, USA) was administered intraperitoneally in various concentrations to four experimental groups, B (5 mg/kg), C (10 mg/kg), D (15 mg/kg), and E (20 mg/kg body weight), while the control group (A) received 100 *μ*l of saline (0.9% NaCl). Behavioural tests were performed 5 min after drug administration, and each behavioural test has been done separately with a 24-hour rest interval between tests in order to minimize animal distress [15].

### 2.2. Behavioural Tests

The behavioural tests used in the study were performed as per the following order.

#### 2.2.1. Marble-Burying Test

Marble test is a simple, inexpensive, and reliable test that measures the compulsive behaviour of burying marbles, which is a common symptom of OCD in rodents. It also allows researchers to test the effects of different drugs or treatments on OCD-like behaviour. When rodents are injected with OCD or anxiety-inducing drugs, the number of marbles buried is expected to increase [16]. The process was as follows: (1) the cage was filled approximately 5-10 cm deep with even surface-wood chip bedding; (2) a regular pattern of six glass marbles was placed on the surface, evenly spaced, and each about 4 cm apart; (3) an animal was placed in each cage and left for 60 min; and (4) the number of buried marbles was counted (to 2/3 their depth) and recorded.

#### 2.2.2. Tail Suspension Test

TST measures the depressive-like behaviour of immobility, which is often associated with OCD in humans and rodents. It also reflects the level of stress and coping ability of the animals. It is widely used, validated, and sensitive to antidepressant drugs.

The tail suspension test (TST) is an experimental method used in scientific research to measure stress in rodents. It is based on the observation that if a rodent is subjected to short-term inescapable stress, then the rodents will become immobile. Each mouse was hung from a tube by its tail for five minutes approximately 10 cm from the ground. During this time, the animal would try to escape and reach for the ground. The time it took each mouse to remain immobile was measured. Each animal was tested only once and out of view of the other animals [17].

#### 2.2.3. Sucrose Preference Test

SPT asses the anhedonic behaviour of reduced preference for sucrose, which is a sign of depression and reduced reward sensitivity in OCD. It also indicates the motivational state and hedonic capacity of the animals. Sucrose preference test is noninvasive and naturalistic test which make it frequently employed as behavioural assessment tool.

Before the test, mice were habituated to the presence of two drinking bottles: one containing 2% sucrose and the other water for four days in their home cage. Following this acclimation, mice had the free choice of either drinking the 2% sucrose solution or plain water for a period of three days. The intake of water and sucrose solution is measured daily, and the positions of two bottles were switched daily to reduce any confounding result caused by a side bias. Sucrose preference was calculated as a percentage of the volume of sucrose intake on the total volume of fluid intake and averaged over the three days of testing [18].

#### 2.2.4. Forced Swim Test

It is a test, centered on a rodent's response to the threat of drowning, whose result has been interpreted as measuring susceptibility to negative mood. It is commonly used to measure the effectiveness of antidepressants as well as the behavioural despair of giving up, which is another indicator of depression and hopelessness in OCD.

Mice were subjected to two trials during which they are forced to swim in an acrylic glass cylinder filled with water and from which they could not escape. The first trial lasted for 15 minutes; then, after 24 hours, a second trial was performed and lasted for 5 minutes. The time that the test animal spent in the second trial without making any movements beyond those required to keep its head above water was measured [19].

#### 2.2.5. Nestlet Shredding

This test evaluates the levels of repetitive compulsive behaviour characteristic of OCD. In addition, it mimics the human compulsions of hoarding or grooming and considered as novel, sensitive, and ecologically valid test. Each mouse was placed in a separate cage containing a single preweighed nestle made of cotton fiber and covered with a filter top. The mouse was left undisturbed in the cage with nestle for 30 min under water and food deprivation. The mouse was then removed and returned to its home cage after the test was complete. The remaining intact nestle material was removed from the cage with forceps, allowed to dry, and weighed [16]. The difference in nestle weight before and after the trial was calculated for each test animal and recorded.

### 2.3. Molecular Tests: Gabra1 and SCL6A4 Gene Expression and Protein Quantification in Mouse Brains

#### 2.3.1. Gene Expression Assessment Using Real-Time PCR

At the end of the behavioural tests, mice were decapitated using a guillotine and their brains were removed, cut into two halves, weighed, snap-frozen using liquid nitrogen, and stored at -80°C for further analysis. Several studies have recommended the use of whole brain for the analysis of serotonin and Gaba as they are both highly expressed in various brain regions related to mood and anxiety [20].

Approximately 50 mg of brain tissue was minced and placed in a labelled conical tube. Total RNA was extracted from brain cells using the Zymo kit (Sigma, St. Louis, MO, USA) and quantitated using nanodrop spectrophotometry (ImplenNp80, 81829, Munchen, Germany). Total RNA was subjected to reverse transcription using the FIRE script Reverse Transcriptase (Sigma, St. Louis, MO, USA). Briefly, 200 ng of extracted RNA was added to 1 *μ*l of 10x RT reaction premix with Oligo (dt) and random primers (Solis BioDyne, Tartu, Estonia), and the mixture was incubated at 65°C for 5 min and cooled to room temperature. The cDNA was quantitated using iTaq Universal SYBR Green PCR (Bio-Rad, CA, USA) and a specific set of primers targeting the serotonin transporter (SLC6A4) and Gabra1 genes (Table 1). The following cycling conditions were adopted: initial denaturation 95°C for 30 sec, followed by 35 cycles of 95°C for 15 sec, 60°C for 1 min, melting curve 65°C for 3 sec, and 95°C for 3 sec. The raw data were exported to Microsoft Excel for analysis [21]. The *ΔΔ*ct method was used to assess the fold increase of the investigated genes against *β*-actin that was used as a housekeeping gene.

#### 2.3.2. Quantitation of Gabra1 and Serotonin Transporter Proteins Using Western Blotting

An amount of 100 *μ*g of proteins from brain homogenate was mixed with 16 *μ*l of Laemmli buffer and 1 *μ*l *β*-mercaptoethanol (Bio-Rad, USA). Each sample was then subjected to 95°C for 5 minutes and loaded onto 14-well polyacrylamide gels (Mini-PROTEAN TGX stain-free gels, Bio-Rad, USA). Gel electrophoresis was performed at 300 V for 20 min, and then, the banded peptides were transferred directly onto nitrocellulose membranes (NCM) (Bio-Rad, Germany). NCMs were blocked with 5% gelatin in Tris-buffered saline (TBS) for 2 hours and then washed two times for 5 min with Tween 20-TBS (TTBS). The NCMs were incubated with the primary antibodies specific for the protein of interest for one hour in 1% gelatin in TTBS. Finally, after washing with TTBS (2 times for 5 min each), NCMs were incubated with secondary antibodies conjugated to horseradish peroxidase in 1% gelatin in TTBS for an hour. The detection of bands was performed using chemiluminescence reagents (Clarity Western ECL Substrate, Bio-Rad, CA, USA), and the intensity of the banded proteins was evaluated using Image Lab software (Bio-Rad, CA, USA). Primary antibodies included in this study were goat anti-mouse Gabra1 antibodies and goat anti-mouse serotonin transporter antibodies [23].

### 2.4. Statistical Analysis

The parameters of the behavioural tests and the fold increase in the expression of the Gabra1 and serotonin transporter genes and protein levels were compared between various treatments using one-way ANOVA followed by Tukey's test for mean separation (SPSS.V.25, IBM, Chicago, Illinois, USA). Significant differences among means were presented at *p* < 0.05.

## 3. Results

### 3.1. Behavioural Tests: OCD-Like and Reward-Related Behaviours in Mice Modulated by RU24969

**Figure 1:**
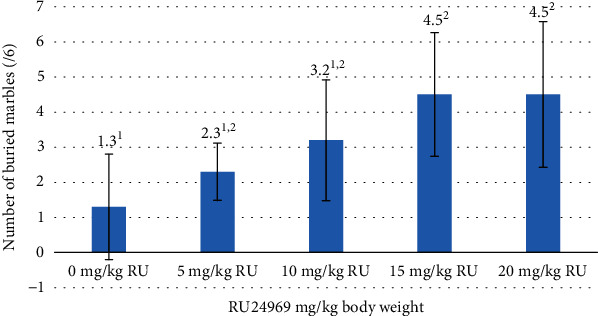
Statistical identification of the number of buried marbles in correlation to the concentrations of RU24969 administered to experimental animals (*n* = 6 mice/group). ^1,2^Means in a column with different numerical superscripts are significantly different (*p* < 0.05).

**Figure 2:**
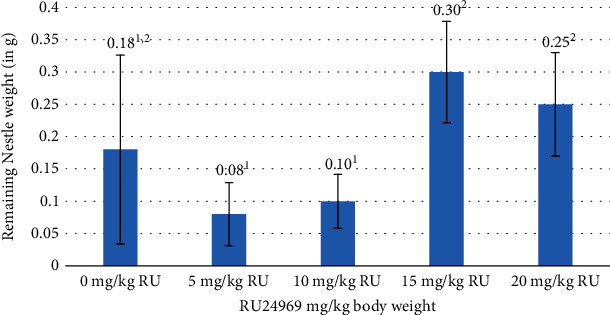
Statistical identification of the remaining nestle weight (g) in correlation to the concentrations of RU24969 administered to experimental animals (*n* = 6 mice/group). ^1,2^Means in a column with different numerical superscripts are significantly different (*p* < 0.05).

**Figure 3:**
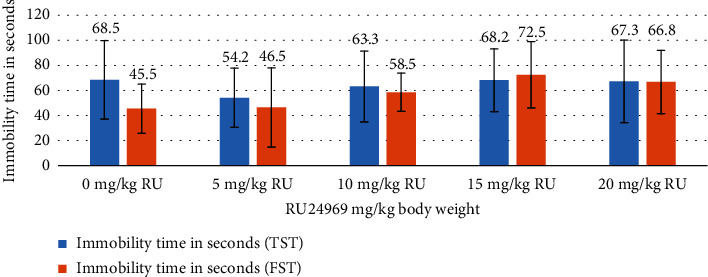
Immobility time (s) for both TST and FST in mice at different doses of RU24969 administration (*n* = 6 mice/group).

The outcomes of the behavioural assessments exhibited divergent results, notably evident in the marble test and the nestlet shredding test, discerned at concentrations of 15 and 20 mg/kg of RU24969 (Figures 1 and 2). Although the forced swim test (FST) and the tail suspension test (TST) did not exhibit statistically significant findings, the most elevated immobility time values were consistently identified at the aforementioned 15 and 20 mg/kg doses of RU24969 (Figure 3).

**Figure 4:**
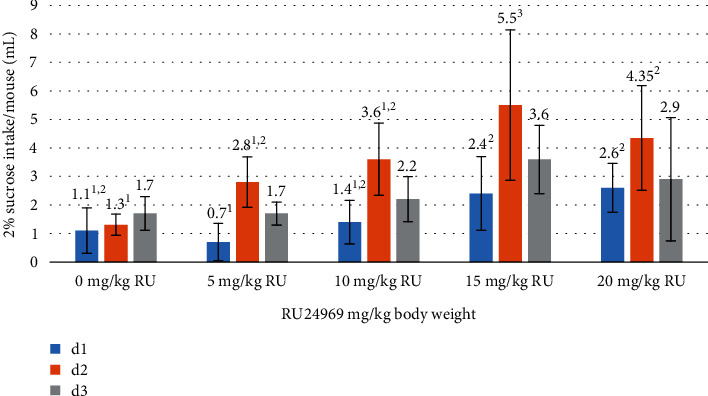
Sucrose intake of mice in various groups included in the study (*n* = 6 mice/group). ^1-3^Means within the same day with different numerical superscripts are significantly different (*p* < 0.05).

**Figure 5:**
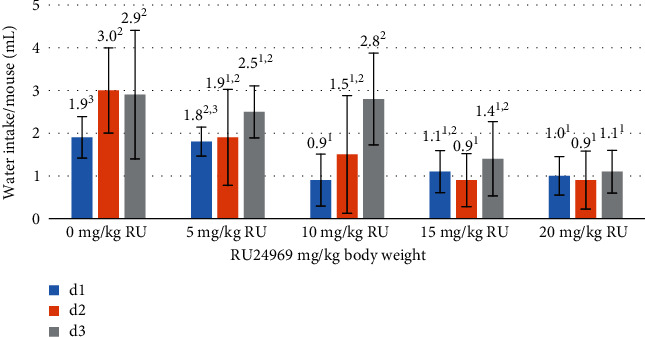
Water intake of mice in various groups included in the study (*n* = 6 mice/group). ^1-3^Means within the same day with different numerical superscripts are significantly different (*p* < 0.05).

Furthermore, it is important to note that the results of the sucrose test revealed a consistent and statistically significant trend that spanned for three consecutive days. Of particular interest is the consistent observation of the highest numerical values, specifically at doses of RU24969 of 15 and 20 mg/kg (Figures 4 and 5). The high sucrose intake observed in the said groups was coupled with significantly low water intake at d1, d2, and d3 in comparison to the control group (1.1, 0.9, and 1.4 ml vs. 1.0, 0.9, and 1.1 ml vs. 1.9, 3.0, and 2.9 ml, respectively) (*p* < 0.05).

### 3.2. RU24969 Differentially Regulates Gabra1 and Serotonin Transporter Gene Expression in Mice

Gabra1 gene expression analysis revealed a significant difference in fold increase/decrease with various doses of RU24969, characterized by a trending event in which values of considerable fold decrease align with the highest two doses of RU24969 15-20 mg/kg of body weight (Table 2).

Regarding serotonin transporter values, no statistically significant findings were depicted. The only discernible pattern is that the lowest value was attributed to group E receiving 20 mg/kg compared to the control (5.28-fold decrease, *p* > 0.05).

### 3.3. RU24969 Decreases Gaba Protein Expression in Mice

Western immunoblotting analysis of Gaba protein revealed a decrement in its signal intensity as the concentration of RU24969 increased, reaching its lowest value at the 15 mg/kg concentration of RU24969 (2.7-fold decrease, *p* > 0.05). On the contrary, serotonin transporter levels did not show a discernible pattern with doses of RU24969 (Table 3).

## 4. Discussion

OCD is marked by continuous unwanted thoughts (obsessions) that are incompatible with one's self-perception and accompanied by intentional actions (compulsions) that appear to have a specific aim [24]. Serotonin, a neurotransmitter renowned for its multifaceted neuromodulator functions, modulates the delicate equilibrium of neural circuits involved in decision-making, impulse regulation, and anxiety processing [25]. Dysregulations within the serotonergic framework have been postulated to trigger the emergence of aberrant behavioural patterns related to obsessive-compulsive disorder (OCD) in murine models [25]. Reduced GABA levels or impaired GABA receptor functioning has been observed in individuals with OCD. This can lead to reduced inhibitory control and contribute to increased anxiety, difficulty with impulse control, and repetitive behaviour seen in OCD [26].

In the current investigation, the compound RU24969 was introduced through intraperitoneal (IP) injection at various dosages of 0, 5, 10, 15, and 20 mg/kg body weight. This administration occurred promptly, in a span of 10 minutes. Subsequently, mice presumptively displaying OCD-like behaviour underwent a battery of five different behavioural tests. The results of these evaluations were consistent with the principal findings established in previous studies.

The marble test, which is often used to assess anxiety-related and OCD behaviour, showed a significant increase in the number of buried marbles in the groups receiving 15 and 20 mg/kg doses of RU24969. This aligns with previous research linking increased marble burial to anxiety-like behaviour, suggesting that these doses may induce anxiety-like responses in mice. However, it is important to note that the marble burial test has been criticized for its lack of specificity and validity as a model of anxiety or OCD, as it can reflect other factors such as defensive or exploratory behaviour [24]. Therefore, the interpretation of the results should be cautious and supported by other behavioural measures. Moreover, the effects of RU24969 on marble burying may depend on the dose and route of administration, as well as the baseline serotonin transporter (SERT) function of the mice [27].

Interestingly, the tail suspension test (TST) and the forced swim test (FST), commonly used to assess depressive-like behaviour and the efficacy of antidepressants, did not exhibit statistically significant differences. However, a consistent trend emerged where the highest values were consistently observed in the 15 and 20 mg/kg dose groups. Although it does not reach statistical significance, this pattern might suggest some potential influence of RU24969 on depressive-like behaviour. Similarly, previous studies have reported that RU24969, an agonist with the same affinity for 5-HT1A and 5-HT1B receptors, significantly increased swimming time in FST, as well as reduced immobility time in TST [28]. These results indicate that RU24969 has antidepressant-like effects mediated by 5-HT1B receptors.

The sucrose preference test (SPT) demonstrated a consistent and significant trend over three consecutive days, mainly in the two highest administrations of RU24969 at doses of 15 and 20 mg/kg. This finding is intriguing, as it could imply a connection between the 5-HT1B agonist and changes in reward-seeking behaviour, potentially indicative of alterations in brain reward pathways. A reduction in sucrose preference indicates a decreased sensitivity to reward. RU24969, by activating the 5-HT1B receptors, could modulate the activity of these pathways and improve the reward value of sucrose [29].

The nestlet shredding test is an additional behavioural assay for evaluating repetitive compulsive behaviour reminiscent of OCD. Here, we did not observe significant changes in nestlet shredding at different doses of RU24969, a serotonin 1A/1B receptor agonist that mimics monoamine dysregulation in OCD [30]. This may suggest that the effects of RU24969 on repetitive behaviour are more context-dependent, requiring further investigation. For example, it is possible that RU24969 affects other aspects of OCD-related behaviour, such as anxiety or cognitive flexibility that are not captured by the nestlet shredding test. Alternatively, it is possible that RU24969 has differential effects on microglial activation and neuroinflammation, which have been implicated in the pathophysiology of OCD [31]. It is worth noting that RU24969 induces hyperlocomotion in rodents [32]. Therefore, the utilization of additional tests such as the open field would have provided more behavioural insights while eliminating any locomotor issues [32].

The real-time PCR results reveal remarkable patterns in gene expression levels in response to RU24969. The Gabra1 gene, which encodes a subunit of the GABA-A receptor, showed a significant decrease in expression as doses of RU24969 increased, reaching the lowest value at the dose of 20 mg/kg. This reduction in Gabra1 expression could be associated with altered GABAergic signaling, which could contribute to observed behavioural changes, such as increased marble burial and anxiety [33].

On the contrary, the expression of the serotonin transporter gene did not exhibit statistically significant changes between the different doses of RU24969. Although unexpected, this finding highlights the complexity of the involvement of the serotonin system in the observed behaviours, suggesting that other factors beyond gene expression may contribute. For instance, it is possible that RU24969 promotes the initiation and propagation of neuroinflammation implicated in OCD pathophysiology [34].

The results of Western blotting provide complementary insights on the protein levels of Gabra1 and serotonin transporter. Interestingly, Gaba protein levels displayed a decrease with increasing doses of RU24969, reaching their lowest point at the 15 mg/kg dose. This corroborates the findings of gene expression and suggests a possible connection between altered Gabra1 expression and observed behavioural responses [35].

On the contrary, serotonin transporter protein levels showed an increase as RU24969 doses increased, with the highest values observed at doses of 5 and 10 mg/kg. The lack of association between serotonin transporter protein levels and RU24969 doses adds a layer of complexity to the study's findings and implies that serotonin signaling may play a role in mediating the behavioural effects of the agonist. Although serotonin is a neurotransmitter involved in OCD and is the target of many pharmacological treatments, the optimal dose and mechanism of action of serotonin reuptake inhibitors for OCD are still unclear [36].

Therefore, our results may provide some clues for further investigation of the role of serotonin in the induction of OCD-like behaviour.

## 5. Conclusion

In conclusion, the results of this study provide valuable information on the potential effects of the 5-HT1B agonist RU24969 in mice, particularly in relation to behaviour associated with anxiety, depression, reward seeking, and repetitive compulsive behaviour reminiscent of OCD. The observed patterns in behavioural tests, gene expression, and protein levels suggest complex interactions between the 5-HT1B receptor system, GABAergic signaling, and serotonin pathways. The results offer an optimized protocol for the induction of OCD-like behaviour in mice, namely, the use of intraperitoneal injection of 15 mg/kg body weight of RU24969 in BALB/c mice.

However, it is important to note that these findings are preliminary and warrant further investigation. The strengths of the study lie in its comprehensive approach, which uses a combination of behavioural tests and molecular analyses to provide a multifaceted understanding of the effects of RU24969. Future research could explore the specific neural circuits and mechanisms through which RU24969 exerts its effects, as well as potential interactions with other neurotransmitter systems.

## Figures and Tables

**Table 1:** The set of primers used in this study [21, 22].

Gene	Accession number	Forward primer	Amplicon length (bp)
Gabra1	XM_017314259	Forward: CCAAGTCTCCTTCTGGCTCAACA Reverse: GGGAGGCAATTTCTGGCACTGAT	111
SLC6A4	NM_010484.2	Forward: GTTGATGCTGCGGCTCAGATCT Reverse: GAAGCTCGTCATGCAGTTCACC	108
*β*-Actin	NM_007393	Forward: AGGCCAACCGTGAAAAGATG Reverse: ACCAGAGGCATACAGGGACAA	101

**Table 2:** Results of RT-qPCR analyses of Gabra and serotonin transporter gene expression in various mouse groups.

Group	Conc. of RU24969 injected (mg/kg)	Number of mice	Gene expression fold increase (+)/decrease (-)	
			Gabra1	Serotonin transporter
A	0	6	1.00	1.00
B	5	6	+1.02^1,2^	-1.88
C	10	6	+1.17^2^	-3.97
D	15	6	-1.01^1,2^	-1.68
E	20	6	-2.35^1^	-5.28
SEM^∗^	—	—	0.081	0.412

^1,2^Means in a column with different numerical superscripts are significantly different (*p* < 0.05). ^∗^SEM = standard error of the mean.

**Table 3:** Variability of Gaba and serotonin transporter concentration as a function of RU doses in various mouse groups.

Group	Conc. of RU24969 injected (mg/kg)	Number of mice	Fold increase (+)/decrease (-) of proteins	
			Gaba	Serotonin transporter
A	0	6	1.00	1.00
B	5	6	-1.20	+1.56
C	10	6	+1.54	+1.08
D	15	6	-2.70	+1.31
E	20	6	-1.90	+1.31
^∗^SEM	—	—	1.078	0.360

^∗^SEM = standard error of the mean.

## Data Availability

All data used to support the findings of this study are included within the article.
